# Impact of aripiprazole discontinuation in remitted major depressive disorder: a randomized placebo-controlled trial

**DOI:** 10.1007/s00213-024-06581-1

**Published:** 2024-03-28

**Authors:** Masahiro Takeshima, Akise Umakoshi, Yuki Omori, Kazuhisa Yoshizawa, Masaya Ogasawara, Mizuki Kudo, Yu Itoh, Naoko Ayabe, Kazuo Mishima

**Affiliations:** 1 Department of Neuropsychiatry, Akita University Graduate School of Medicine, Akita, Japan; 2 Department of Psychiatry, Tokyo Metropolitan Institute for Geriatrics and Gerontology, Tokyo, Japan; 3 Department of Regional Studies and Humanities, Faculty of Education and Human Studies, Akita University, Akita, Japan

**Keywords:** Aripiprazole, Augmentation, Depression, Maintenance, Randomized controlled trial

## Abstract

**Rationale:**

The efficacy and safety of antidepressant augmentation therapy with aripiprazole (AATA) has been established; however, the ongoing effects of continuing aripiprazole after remission remain unclear because no studies have examined this issue.

**Objectives:**

We aimed to explore the effect of AATA discontinuation on the major depressive disorder (MDD) recurrence risk in patients with remitted MDD after AATA.

**Methods:**

This 24-week, multicenter, placebo-controlled, double-blind, randomized trial evaluated recurrence risk in patients with MDD who achieved remission with AATA. Differences in MDD recurrence, as defined by the Diagnostic and Statistical Manual of Mental Disorders, Fifth Edition, between the two groups were compared using survival analysis. The differences in depressive symptom severity and social functioning between the two groups were compared using a mixed model with repeated measures. Extrapyramidal symptoms and akathisia were also assessed.

**Results:**

Twenty-three participants were randomized and treated. Two patients in each group experienced recurrence during the study. Kaplan–Meier analysis with Log-rank comparison showed no difference in recurrence between groups (*p* = 0.642). No significant difference in interactions between group and period was observed in the 17-item Hamilton depression rating scale (*p* = 0.492) or the Social and Occupational Functioning Assessment Scale (*p* = 0.638). No patients developed extrapyramidal symptoms or akathisia.

**Conclusions:**

Definitive conclusions could not be drawn owing to the small sample size. This study represents a starting point for investigating the safety of aripiprazole discontinuation on recurrence in patients with MDD who have achieved remission with AATA. Future studies with appropriate sample sizes calculated based on this study are needed.

## Introduction

Antidepressant augmentation therapy with aripiprazole (AATA) is a valuable treatment option for major depressive disorders (MDDs) (Mohamed et al. 2017; Lewis and Lewis 2023; McIntyre et al. 2023). A recent network meta-analysis reported that AATA increased remission and response rates by approximately 1.8-fold and was comparable in tolerability to placebo (Yan et al. 2022). In addition, a meta-analysis of long-term open-label studies reported the efficacy of AATA in refractory MDD, particularly at doses below 5 mg, and increasing treatment benefits with increasing duration of the treatment (Seshadri et al. 2021). Guidelines recommend AATA for partial or no response to antidepressants (Cleare et al. 2015; Kennedy et al. 2016; Malhi et al. 2021; Seo et al. 2021).

While the efficacy of AATA is well established, the maintenance effect of AATA in patients whose MDD has remitted with AATA remains unclear. This lack of clarity exists because no randomized controlled trials to date have examined the harms and benefits of continuing or discontinuing aripiprazole in patients with MDD who have achieved remission with AATA. Aripiprazole is considered safe among the atypical antipsychotics (Yan et al. 2022). However, evidence regarding the safety of long-term AATA treatment is insufficient. A meta-analysis of long-term, open-label studies reported significant weight gain in 25–28%, akathisia in 15–16%, and tardive dyskinesia in less than 1% of patients treated with AATA. However, because this meta-analysis does not include randomized controlled trials, whether aripiprazole increases adverse events in long-term AATA treatment remains unclear. In addition, the studies included in the meta-analysis lasted up to 52 weeks (Seshadri et al. 2021; Berman et al. 2011; Kamijima et al. 2018). Continuation of AATA may cause tardive dyskinesia because mood disorders and long-term use of antipsychotics are risk factors for tardive dyskinesia (Solmi et al. 2018; Frei 2019). In addition, the cost of maintenance therapy with AATA is an important issue. Previous studies have reported that augmentation with aripiprazole is more cost-effective than augmentation with olanzapine or quetiapine (Saylan et al. 2013) and more cost-effective than switching to bupropion in patients with MDD responding insufficiently to antidepressant treatment (Yoon et al. 2018). However, prior studies have investigated the cost-effectiveness of AATA for acute MDD, but not for maintenance treatment. The number of patients with MDD continues to increase, along with the associated costs (Greenberg et al. 2021); therefore, clarifying the usefulness of maintenance therapy with AATA could help reduce medical costs. Therefore, there is an urgent need to evaluate the harms and benefits of discontinuing AATA in remitted patients.

This exploratory study aimed to determine the effect of AATA discontinuation on the risk of MDD recurrence in patients whose MDD has remitted with AATA.

## Materials and methods

### Study design and procedure

This randomized, double-blinded, parallel-group, placebo-controlled, 24-week, multicenter study explored if discontinuing aripiprazole increases the risk of recurrence in patients with MDD in remission after AATA. This study was conducted at two sites in Akita Prefecture, Japan, between November 28, 2017, and November 27, 2023. The study was originally designed to last for 5 years but was extended by 1 year due to a shortfall in enrollment. This study is not pre-registered anywhere. Participants were recruited through their outpatient psychiatrists and flyers. Patients were screened for eligibility for up to 4 weeks. The study psychiatrist made the final decision on the eligibility by interviewing or examining the patient and assessing the 17-item Hamilton Depression Rating Scale (HAM-D17) total scores and reviewing the electronic medical records (Tabuse et al. 2007).

### Patients

Adult men and women (≥ 20 years of age) meeting the Diagnostic and Statistical Manual of Mental Disorders, Fifth Edition (DSM-5) diagnostic criteria for MDD who achieved full remission and maintained full remission for 20 weeks to 2 years after AATA were eligible for inclusion in this study (American Psychiatric Association 2013). In the DSM-5, full remission is defined as the absence of significant impairment or signs of MDD in the past 2 months. However, mild symptoms such as mild depressive mood for less than 3 days per week was not considered a severe symptom in patients in this study. The duration of this sustained remission was established based on discussions among the investigators. The duration of remission was determined based on patient interviews and electronic medical records, not the HAM-D17. In addition, participants were required to have a HAM-D17 total score ≤ 7 and continue receiving the same dose of antidepressants and aripiprazole that they were taking during and after remission to be eligible for study enrollment.

Patients were excluded if they had a history or current diagnosis of psychotic disorder, bipolar disorder, borderline personality disorder, eating disorder, or substance or alcohol use disorder. In addition, patients < 20 years of age, patients lacking capacity to consent, pregnant or lactating women, participants with hypersensitivity to aripiprazole and adrenalin, and those with suicidal ideation were ineligible. Rarely, suicidal ideation persists after remission in patients with MDD (Nierenberg et al. 2010). Such patients may have been a heterogeneous group compared to those who were not, which may have influenced the results of this study. Therefore, patients with suicidal ideation were excluded in this study. Patients were also excluded if their antidepressant dosage had changed since the start of AATA or if antidepressants or antipsychotics that they were not taking at the start of AATA were added.

### Randomization and blinding

Eligible patients were randomized (1:1) to receive either aripiprazole powder (prescribed dose in remission) or a placebo (lactose) once daily based on a computer-generated randomization schedule. Both the patients and the treating and evaluating psychiatrists were blinded. Aripiprazole powder and placebo was packaged in the same bag and indistinguishable from aripiprazole by appearance, color, and smell. All patients continued to receive baseline psychotropic medications at the same dose, without changes, and at the same time as before entering the study. Patients were prohibited from adding psychotropic medications not taken at baseline or increasing the dose of psychotropic medications taken at baseline.

### Outcome measures

The following information was collected at baseline: age, sex, history of education, marital status, cohabitants, details of psychotropic medications used, age at MDD onset, lifetime number of major depressive episodes, time from the current aripiprazole dose to remission, and duration of remission to baseline. Patients were followed up for 24 weeks (at 0, 2, 4, 8, 12, 16, 20, and 24 weeks) to assess the primary and secondary endpoints. All outcomes were evaluated by psychiatrists skilled in clinical research and clinical trials. The primary endpoint was the recurrence of MDD according to DSM-5 (18) (American Psychiatric Association 2013). “Relapse” is not mentioned in the DSM-5, whereas a recurrent episode is defined in the DSM-5 as a return of symptoms during a remission. Therefore, the term “recurrence” was used in this study rather than “relapse.” The secondary endpoints were changes in depressive symptoms and social functioning from baseline to 24 weeks. The HAM-D17 (Tabuse et al. 2007) was used to assess depressive symptom severity, and the Social and Occupational Functioning Assessment Scale (SOFAS) was used to assess social functioning (Inada and Kunihiro Iwamoto 2016). Tardive dyskinesia, dystonia, akathisia, and Parkinsonism were assessed based on the interview, observation, and physical examination without rating scales at each visit.

### Statistical analyses

The sample size for this study was set at 30 without sample size calculation because of the lack of similar previous studies and the study’s exploratory nature. Non-normally distributed continuous and categorical variables are expressed as medians and interquartile ranges and numbers (%), respectively. The Mann–Whitney U test was used for continuous variables, and a chi-square or Fisher’s exact test was used for categorical variables to examine the differences between the aripiprazole and placebo groups. The event-free survival duration was determined from the allocation date to MDD recurrence. Further subgroup analyses were performed, dividing patients into those who recovered (more than 24 weeks after remission) and those who remitted but did not recover (20 to 23 weeks after remission) according to previous studies (Van Londen et al. 1998; Kato et al. 2021). Cumulative incidence curves were derived on intention-to-treat analysis using the Kaplan–Meier method, and differences between the two groups were analyzed using the Log-rank test. A mixed-effects model for repeated measures (MMRM) was used to compare the severity of depressive symptoms and social function between the two groups during the 24-week study period. MMRM has been widely used to effectively avoid bias due to missing data, a common problem in longitudinal clinical trials. Statistical analyses were performed using SPSS Statistics 28.0 (IBM Corp. Armonk, NY, USA). Statistical significance was set at *p* < 0.05 (two-sided).

## Results

**Fig. 1:**
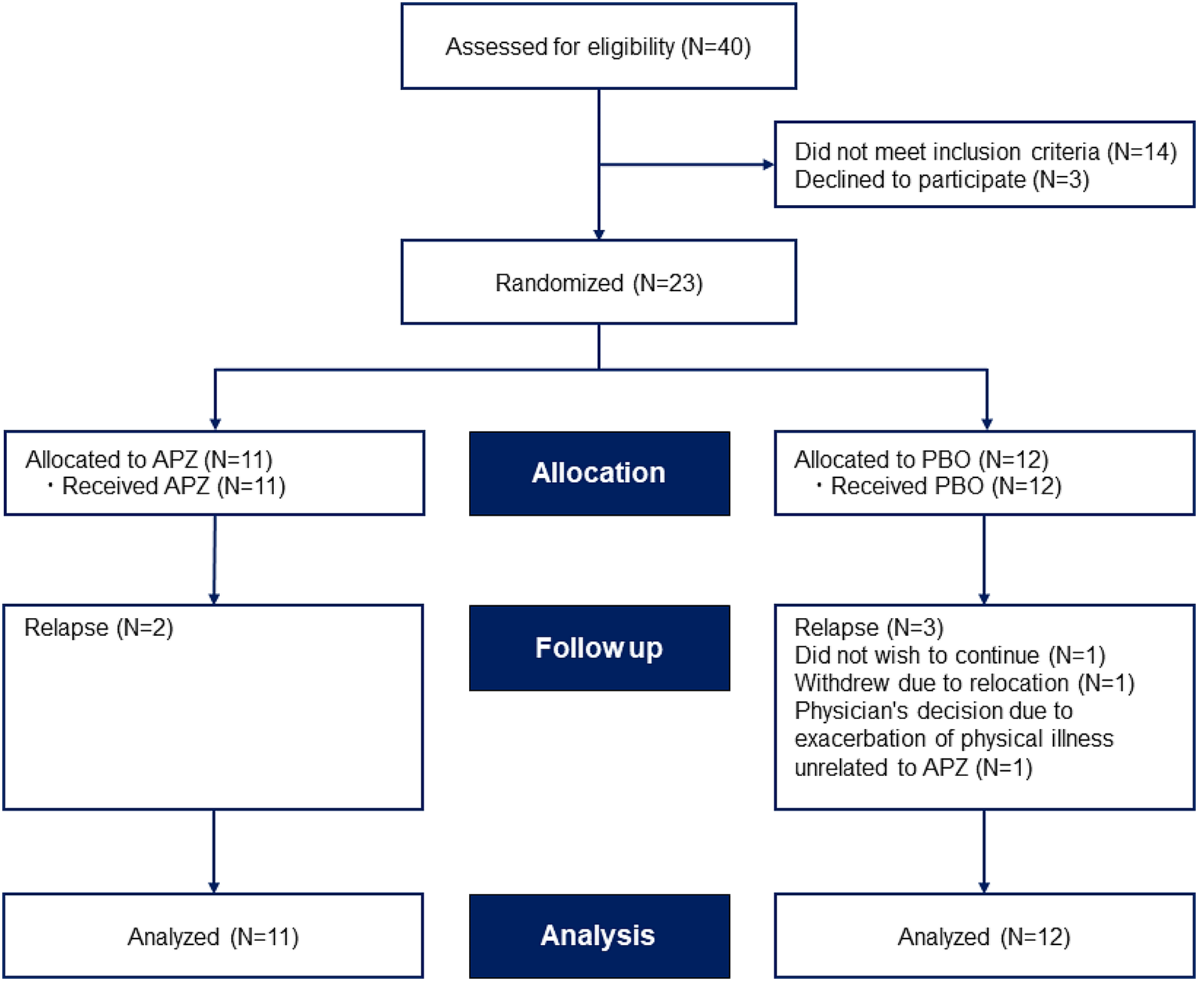
CONSORT diagram. APZ, aripiprazole; CONSORT, Consolidated Standards of Reporting Trials; PBO, placebo

The target sample size of 30 was not achieved with 21 participants enrolled during the pre-planned 5-year study period. The study was extended by 1 year, but only two additional participants were enrolled. The study was not extended further due to lack of funds and manpower. Twenty-three patients participated in the study, and all of them were prescribed aripiprazole tablets as augmentation therapy prior to randomization, and none had ever been prescribed aripiprazole powder. No participants were prescribed antipsychotics other than aripiprazole at baseline. All participants were randomized and received treatment, and 15 (65.2%) of the randomized participants completed the trial (Fig. 1). Two patients in the aripiprazole group recurred at weeks 4 and 20, and three in the placebo group recurred at weeks 4, 8, and 16, respectively. All recurrent patients were recovered patients who had been in remission for at least 24 weeks at baseline. In addition, three participants dropped out without recurrence in the placebo group; one who relocated dropped out, one withdrew from the study, and one dropped out because of exacerbation of a comorbid physical illness unrelated to the study, according to the physician’s decision. All patients with recurred MDD refused to be evaluated after the recurrence, as specified in the protocol of this study. No data before recurrence or dropouts were missing.

The participant characteristics are shown in Table 1. The participants were mostly older, with a median age of 69 years (first quartile, 52–third quartile, 75 years), and 56.5% were women. Nineteen patients received antidepressant monotherapy, and 4 received a combination of two antidepressants. Aripiprazole doses were 3 mg/day in 19 patients and 6 mg/day in 4, with an average of 3.5 mg/day. At baseline, participants received AATA for a median of 33 weeks (first quartile, 25–third quartile, 49 weeks) and had been in remission for a median of 27 weeks (first quartile, 21–third quartile, 44 weeks). There were no differences between the two groups in terms of demographics, dose of aripiprazole, severity of depressive symptoms, social function, concomitant psychotropic drugs other than antidepressants and aripiprazole, or at baseline.

**Table 1:** Demographic and clinical characteristics of the participants at baseline

	APZ (*N* = 11)	Placebo (*N* = 12)	P value
Age, years	71 (58, 79)	64 (51.25, 72.5)	0.211
Female^a^	5 (54.5%)	5 (41.7%)	1.000
Married or cohabitants^a^	11 (100%)	11 (91.7%)	1.000
Education ^b^			0.472
< High school	1 (9.1%)	2 (16.7%)	
High school diploma	8 (72.7%)	10 (83.3%)	
Associate degree	1 (9.1%)	0 (0%)	
Bachelor’s degree	1 (9.1%)	0 (0%)	
Onset of MDD (age, years)	66 (47–77)	61.5 (45.75–69)	0.487
Past major depressive episodes^a^			0.827
1	4 (36.4%)	5 (41.7%)	
2	4 (36.4%)	5 (41.7%)	
3 or more	3 (27.3%)	2 (16.7%)	
Time from aripiprazole augmentation therapy to remission (weeks)	4 (2–8)	5.5 (4.0–8.0)	0.695
Time from remission to assignment (weeks)	26 (21–48)	29 (20.25–43)	0.833
Dose of APZ (mg/day)	3 (3–3)	3 (3–6)	0.19
Number of ADs			0.317
1	8 (72.7%)	11 (91.7%)	
2	3 (27.3%)	1 (8.3%)	
Lithium^a^	1 (9.1%)	0 (0%)	0.478
Hypnotic^a^	7 (63.6%)	9 (75.0%)	0.444
Anxiolytics^a^	3 (27.3%)	2 (16.7%)	0.64
HAM-D17	0 (0–4)	1 (0–2)	0.786
SOFAS	80 (80–90)	90 (80–97.5)	0.379

*Notes*: Values are presented as medians (interquartile ranges) or numbers [%]

*Abbreviations*: AD, antidepressant; APZ, aripiprazole; HAM-D17, 17-item Hamilton Depression Rating Scale; MDD, major depressive disorder; SOFAS, Social and Occupational Functioning Assessment Scale

^a^Fisher’s exact test

^b^Chi-square test

**Fig. 2:**
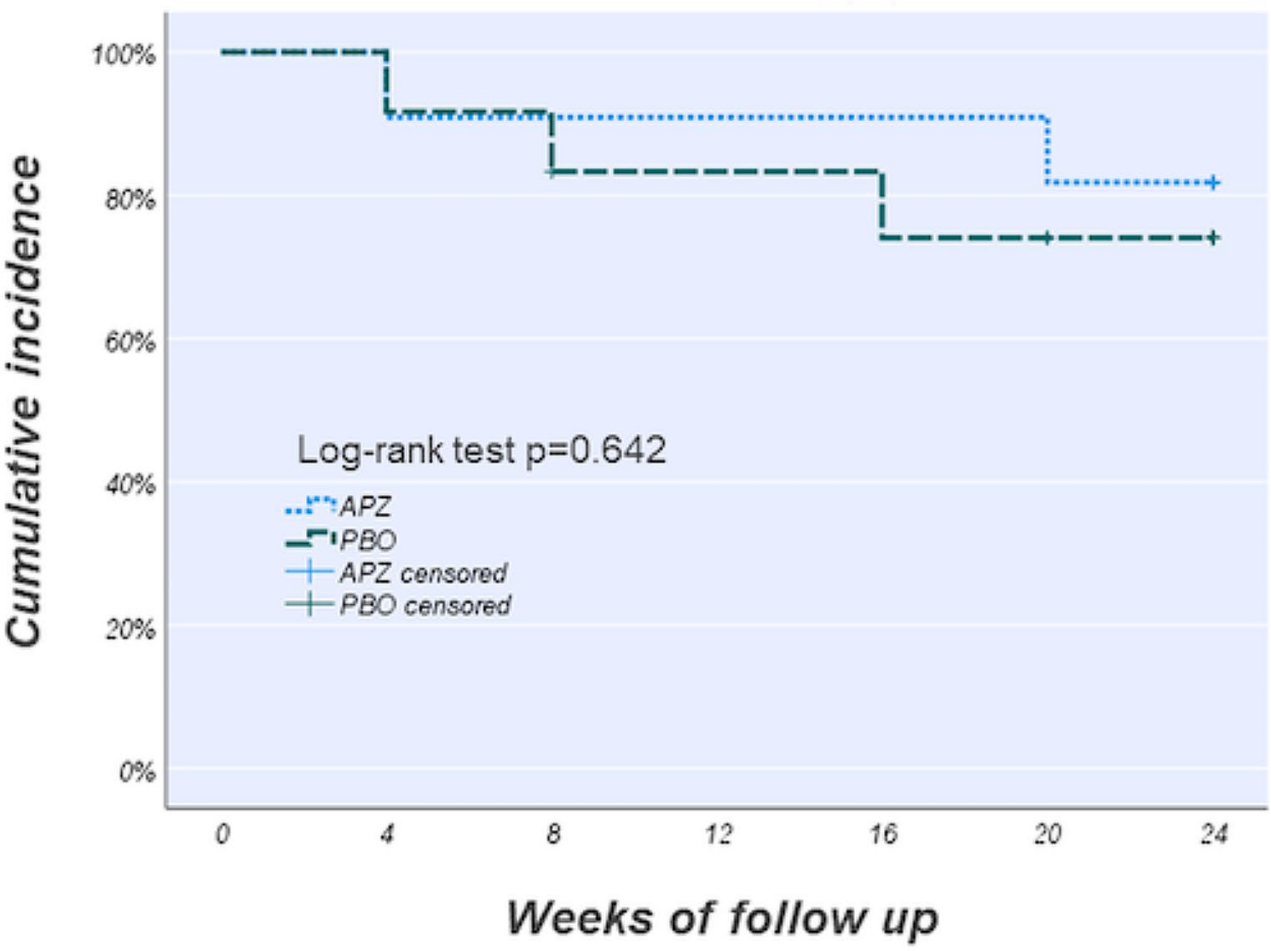
Kaplan–Meier survival curves for time to recurrence. The green dashed line indicates the aripiprazole group (*N* = 11), and the blue dotted line indicates the placebo group (*N* = 12). APZ, aripiprazole; PBO, placebo

During the study period, 18.2% of the patients in the aripiprazole group and 25.0% of those in the placebo group recurred. The cumulative incidence of MDD recurrence in the aripiprazole and placebo groups is shown in Fig. 2. Kaplan–Meier analysis showed no difference in MDD recurrence between the two groups (Log-rank *p* = 0.642). In patients who met the criteria for recovery at baseline, MDD recurred in 28.6% (2/7) of the patients in the aripiprazole group and 37.5% (3/8) of those in the placebo group. A subgroup analysis of 15 patients in the recovery group showed no significant difference in recurrence between the two groups (Log-rank *p* = 0.622).

**Fig. 3:**
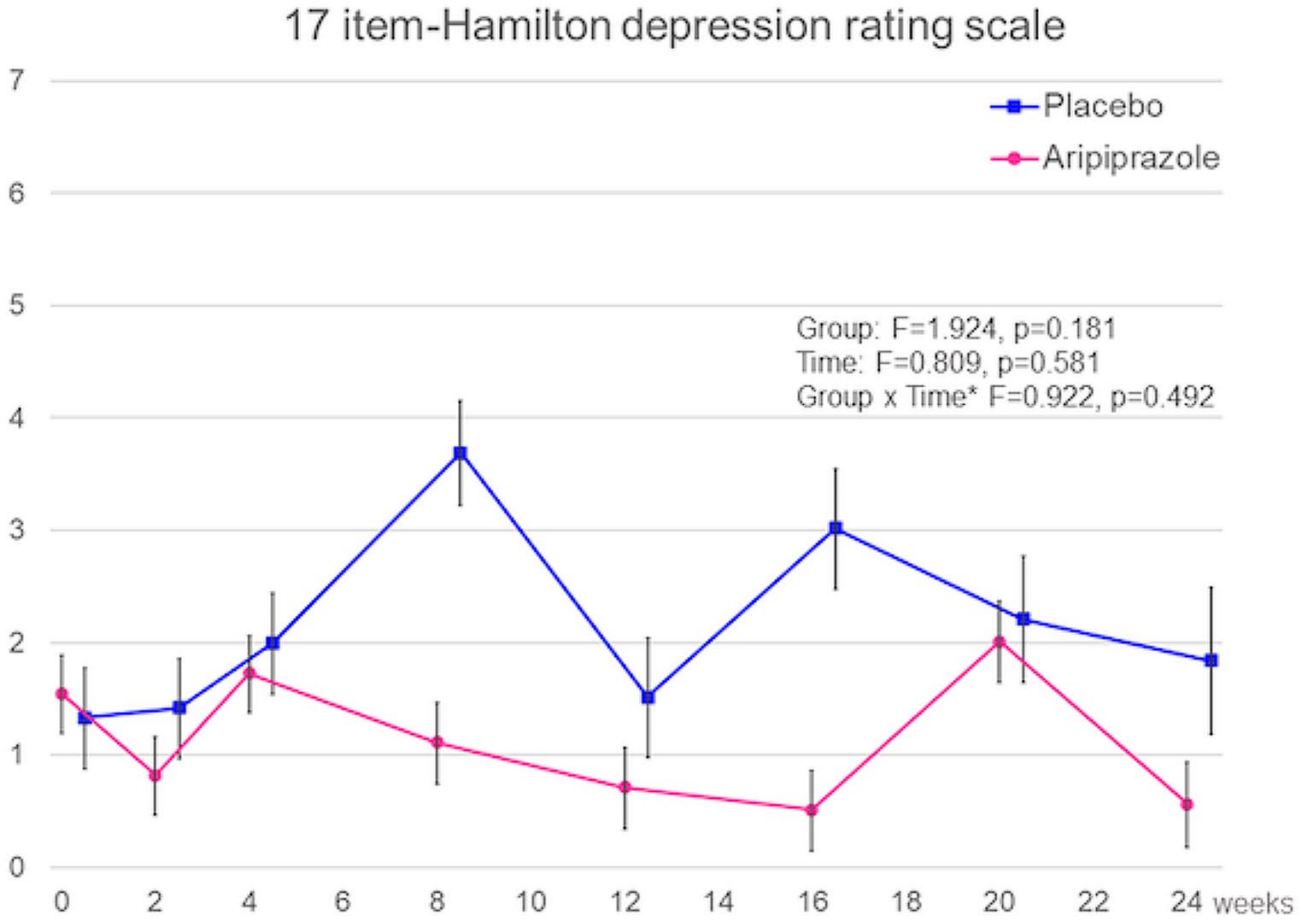
Comparison of changes in severity of depressive symptoms in the aripiprazole and placebo groups. *Note*: Bar indicates standard error

**Fig. 4:**
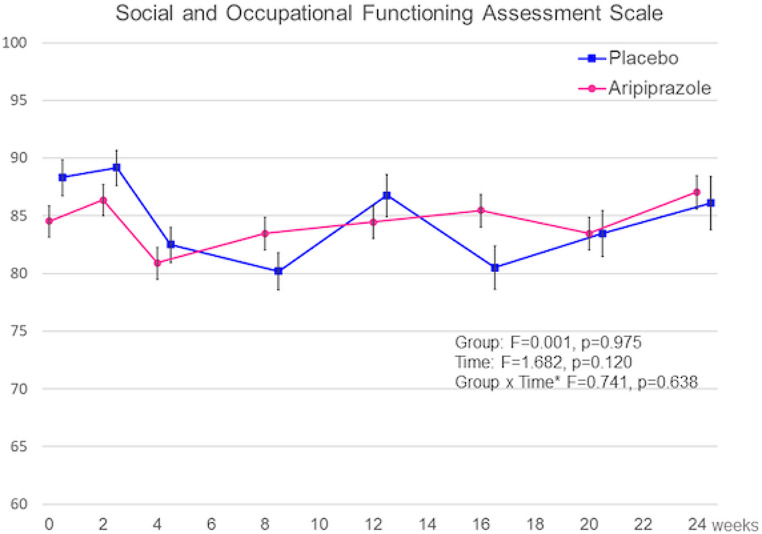
Comparison of changes in social function in the aripiprazole and placebo groups. *Note*: Bar indicates standard error

Figures 3 and 4 show the results of the MMRM, including group effects, time effects, and the interaction of time-by-group effects, to present the effects of AATA discontinuation on the severity of depressive symptoms and social function in patients with remitted AATA. No significant difference in the interaction between group and period was observed for HAM-D17 (F = 0.922, *p* = 0.492) or SOFAS (F = 0.741, *p* = 0.638).

Regarding safety, no patient developed parkinsonism, akathisia, tardive dyskinesia, or dystonia during the study period.

## Discussion

This study is the first exploratory randomized controlled trial to evaluate the harm and benefits of aripiprazole discontinuation in patients whose MDD remitted after AATA. There were no significant differences in the efficacy and safety measures between the aripiprazole continuation and placebo groups.

Furthermore, no significant differences were found in the recurrence of MDD, severity of depressive symptoms, or social functioning between the aripiprazole continuation and discontinuation groups. In a previous randomized controlled trial in which patients with MDD who responded to a olanzapine/fluoxetine combination (OFC) were randomized to continued OFC or fluoxetine alone for up to 27 weeks, the time-to-relapse was significantly longer in the OFC group than in the fluoxetine group (Brunner et al. 2014). The reasons for the differences in results between this study and the previous study by Brunner et al. (2014) remain unclear. The different pharmacological profiles of aripiprazole and olanzapine, different inclusion criteria between studies (we included patients in remission with augmentation therapy, whereas Brunner et al. included those who had failed treatment with two or more different antidepressants but responded to OFC), and differences in patient demographics (our participants were older and had a smaller proportion of female participants, fewer major depressive episodes, and a lower age of MDD onset than those in the previous study) may have contributed to the difference in results. In addition, patients in the present study received a median of 33 weeks of AATA at baseline compared to 12 weeks of OFC in the previous study, which may have influenced the difference in results between the studies. However, because of the very small sample size and lack of power, no conclusions could be drawn from this study. Therefore, a more sophisticated study design with a larger cohort and more representative patients is needed.

No antipsychotic-induced side effects such as tardive dyskinesia, dystonia, akathisia, or Parkinsonism were observed in this study. In previous open-label studies of aripiprazole, tardive dyskinesia occurred in 0.4% of patients with MDD in a 52-week study with an average dose of approximately 10 mg/day. No tardive dyskinesia occurred in a 52-week study with an average dose of approximately 3.5 mg/day (Berman et al. 2011; Kamijima et al. 2018). The risk factors for tardive dyskinesia include older age, mood disorders, and cumulative antipsychotic doses (Solmi et al. 2018; Frei 2019). Although most participants in this study were older patients with MDD, the low cumulative antipsychotic dose and short duration of the study may explain why none developed tardive dyskinesia. It could be specified that the absence of tardive dyskinesia could be due not only to the low dose of aripiprazole but also to the short duration of the study, which is one of its limitations as stated below. Acute akathisia and dystonia are more likely to occur early in the course of antipsychotic therapy (Taylor et al. 2021). Understandably, patients who had been on low-dose aripiprazole for a relatively long period of time (median 30 weeks) at baseline, but did not have extrapyramidal symptoms, did not develop these symptoms during the short 24-week study period.

This study had several limitations. First, this was a small exploratory study at the time of study design, and required sample size could not be reached, resulting in insufficient power. Second, the participants in this study, who were primarily older individuals at high risk for recurrence of MDD (Mueller et al. 2004; Mitchell and Subramaniam 2005), were not a representative sample of the general population. Third, the short duration of this study (24 weeks) precluded long-term evaluation of the effects of aripiprazole discontinuation in patients who had remitted with AATA. Fourth, because the participants in this study were those who had been in remission for at least 20 weeks, it is unclear whether the results of this study apply to those who have been in remission for a short time. Fifth, this study did not evaluate cost-effectiveness. Therefore, the increased economic burden of maintenance therapy with AATA could not be assessed.

In conclusion, although this study indicates that patients with MDD in remission for at least 20 weeks with AATA do not have a significantly increased risk of recurrence after AATA discontinuation, no definitive conclusions could be drawn because of limitations such as a small sample size. This study provides a notable starting point for studying the safety of aripiprazole discontinuation on recurrence safety in patients with MDD who have achieved remission with AATA. Furthermore, our findings may enrich the discussion on the need for long-term pharmacological therapy in patients who have successfully overcome a depressive episode. Therefore, further studies are needed.

## Data Availability

Data sharing is applicable if the corresponding author determines that the reason is appropriate.
